# Automated Tissue Classification Framework for Reproducible Chronic Wound Assessment

**DOI:** 10.1155/2014/851582

**Published:** 2014-07-08

**Authors:** Rashmi Mukherjee, Dhiraj Dhane Manohar, Dev Kumar Das, Arun Achar, Analava Mitra, Chandan Chakraborty

**Affiliations:** ^1^School of Medical Science & Technology, Indian Institute of Technology, Kharagpur, West Bengal 721302, India; ^2^Department of Dermatology, Midnapore Medical College Hospital, Midnapore, West Bengal 721101, India

## Abstract

The aim of this paper was to develop a computer assisted tissue classification (granulation, necrotic, and slough) scheme for chronic wound (CW) evaluation using medical image processing and statistical machine learning techniques. The red-green-blue (*RGB*) wound images grabbed by normal digital camera were first transformed into *HSI* (hue, saturation, and intensity) color space and subsequently the “*S*” component of *HSI* color channels was selected as it provided higher contrast. Wound areas from 6 different types of CW were segmented from whole images using fuzzy divergence based thresholding by minimizing edge ambiguity. A set of color and textural features describing granulation, necrotic, and slough tissues in the segmented wound area were extracted using various mathematical techniques. Finally, statistical learning algorithms, namely, Bayesian classification and support vector machine (SVM), were trained and tested for wound tissue classification in different CW images. The performance of the wound area segmentation protocol was further validated by ground truth images labeled by clinical experts. It was observed that SVM with 3rd order polynomial kernel provided the highest accuracies, that is, 86.94%, 90.47%, and 75.53%, for classifying granulation, slough, and necrotic tissues, respectively. The proposed automated tissue classification technique achieved the highest overall accuracy, that is, 87.61%, with highest kappa statistic value (0.793).

## 1. Introduction

Globally, chronic wounds (CWs) are a major threat to the public health and economy since they have detrimental effect on patient's quality of life causing depression, social separation, and high costs for treatment. CWs are those that did not proceed through an orderly and timely reparative process to produce anatomic and functional integrity of the injured site, generally remaining unhealed for longer than 6 weeks [1]. Furthermore, the healing process may be delayed if appropriate treatment is not provided based on accurate diagnosis. In the United States, CW affects approximately 6.5 million patients [2]. The prevalence of CW was 4.48 per 1000 of a study population in India [3]. Thus, it can be very well-understood that this type of health threat is progressively increasing as a silent epidemic. Although diabetic foot ulcers, venous ulcers, and pressure ulcers are considered as the 3 main types of CW, still, burn, malignant ulcer, and* Pyoderma gangrenosum* incidence rate is quite significant [2]. These CWs are often resistant to healing and require long term medical care.

Quantitative assessment of CW still depends on visual inspection and manual techniques for depicting the shape of the wound perimeter, surface, depth, and so forth and the biological nature of the skin tissues percentage of each class, wound severity stage, burn degree, and so forth. CW includes mainly three types of tissues namely, nonuniform mixture of yellow slough, black necrotic, and red granulation tissues. These tissue types play a pivotal role in understanding the healing progress of different CWs. However, during CW diagnosis, clinicians frequently face difficulty in estimating percentage of these tissues within wound area due to color ambiguities. Routine diagnosis of CW completely relies on clinician's (namely, burn unit nurse practitioners/dermatologists) manual examination which involves measurement methods like ruler-based methods, transparency tracing, alginate casts, and so forth [4]. Similarly, assessing the type and proportion of tissues remains highly debatable as assessment is performed visually and then recorded on a red-black-yellow scale corresponding, respectively, to the dominant color of the different tissues found on a wound that is, granulation, slough, and necrosis. These conventional processes are very often inaccurate since such evaluation depends on his/her clinical experience and causes extreme discomfort to the patients. Moreover it is time consuming and expert-intensive. In order to provide an efficient as well as rapid CW diagnosis scheme, wound tissue classification is indeed required so that the percentage of tissues can be quantitatively estimated on regular basis for healing rate assessment during the treatment. The principal objective of the present study is to develop an automated tissue classification scheme for reproducible CW assessment using computer vision and machine learning methods. These techniques can serve as effective tools for precise wound bed demarcation, segmentation, and tissue identification in CWs.

There have been efforts to use image processing techniques for automatic and quantitative analysis of CW images. Leg ulcer regions were identified using contour detection with histogram segmentation [5] and active contour modeling [6, 7]. Zhang et al. applied region growing method for edge detection on digitized skin tumor images [8]. Attempt was made to extract wound region using texture analysis [6, 9]. Wound tissues were characterized using different algorithms, namely, histogram thresholding, mean shift smoothing, region growing, and graphs [10, 11]. A case-based tissue classification approach was developed for monitoring leg ulcer healing [12]. Pressure ulcer lesion area was evaluated under telemedicine system [13]. Serrano et al. developed a computer aided diagnostic process for evaluating burns by depth of injury [14]. A supervised tissue classification from color images was designed for assessment of wound lesions [15].

**Figure 1:**
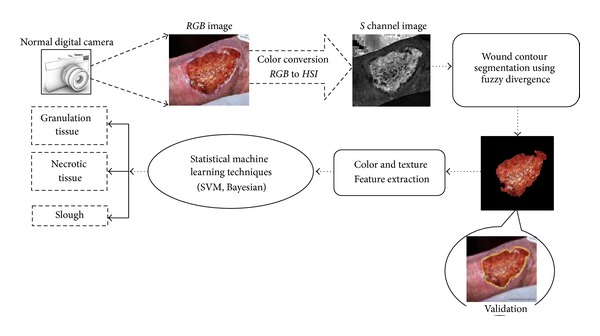
Work flow of the proposed computer assisted imaging tissue classification technique.

In India, very few studies have been performed though risk factors of CW like diabetes, atherosclerosis, tuberculosis, leprosy, and trauma are very much prevalent. Nayak et al. addressed the composition of different types of tissue based on color and pigmentation inside the wound by image processing [16]. Extensive literature survey revealed that there is an urgent requirement for quantitative estimation of wound tissue classification within the wound bed, which might assist clinicians to effectively monitor the wound healing rate. In view of this, we have proposed here a computer assisted tissue classification methodology using fuzzy divergence based CW region segmentation and statistical machine learning techniques. The overall workflow has been depicted in Figure 1.

## 2. Methodology

### 2.1. Selection of Wound Images and Preprocessing

**Figure 2:**
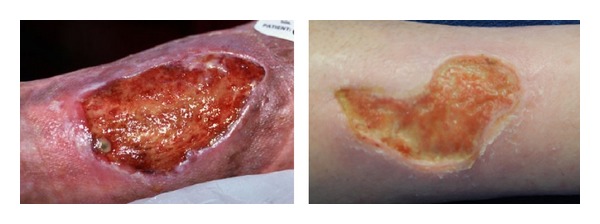
Photographs of chronic wounds grabbed by a digital camera.

In the present study, CW images from Medetec medical image database [17] (http://www.medetec.co.uk/files/medetec_image_databases.html) were considered which were captured under same optical imaging set-up. Normal digital optical camera was used by the dermatologist to grab images (*R*: red; *G*: green; *B*: blue, i.e., *RGB* format) from wound sites of CW patients.  Figure 2 shows two representative CW images.

In the present study, we have considered all the six types of CW, namely, burn, diabetic ulcer, malignant ulcer,* Pyoderma gangrenosum*, venous ulcer, and pressure ulcers. These wounds are mainly characterized by the target clinical parameters, namely, granulation, slough, and necrotic tissues [18]. In fact, the percentage of each type of tissues plays major role in evaluating various chronic wounds. The granulation tissue comprises new connective tissue and tiny blood vessels that form on the surfaces of a wound during the healing process. It looks light red and/or dark pink in color. Necrotic tissue is basically dead tissue that generally results from an inadequate local blood supply. It is of black color and found in a wide variety of wound types, including burns and all types of chronic wounds. In contrast, slough is a yellow fibrinous tissue that consists of fibrin, pus, and proteinaceous material. It can be found on the surface of a previously clean wound bed and it is thought to be associated with bacterial activity. The accumulation of necrotic tissue or slough in a chronic wound is of major clinical significance [18], because it is thought to promote bacterial colonization and prevent complete repair of the wound. Here, we considered 222 regions as granulation tissue, 451 regions as slough tissue, and 94 regions as necrotic tissue based on 74 wound images (burn (*n* = 12), diabetic ulcer (*n* = 24), malignant ulcer (*n* = 14),* Pyoderma gangrenosum* (*n* = 8), venous ulcer (*n* = 7), and pressure ulcer (*n* = 9)) identified by medical experts from the wound database.

Due to rapid photography and dust attached to the camera lens, the wound images are very often affected by salt and paper noise. In order to remove this noise from wound images, median filter [19] being the most popular nonlinear filter was here used by replacing the window center value by the median value of center neighborhood. In fact, the median filter using a 5 × 5 structural element window was used to *RGB* channels individually for wound images. It basically maintains the edges of the wound areas and reduces the noise. In effect, the CW images become more homogeneous leading to improved wound bed segmentation.

### 2.2. Color Space Conversion from *RGB* to *HSI*

The grabbed wound images were in fact color images in *RGB* format. Generally, clinicians/nurse practitioners at burn unit face diagnostic problem due to color nonuniformity present in *RGB* wound images. In addition, since *RGB* components are highly interrelated, it is not proper to use chromatic information directly.

**Figure 3:**
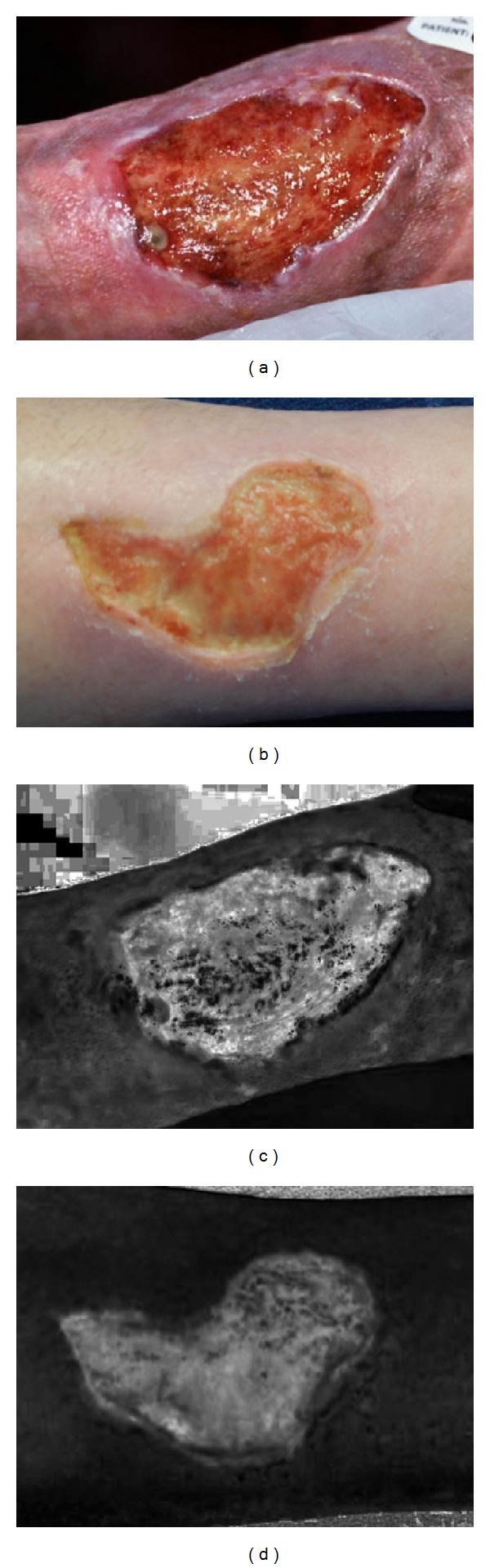
Color conversion: (a-b) original *RGB* images; (c-d) *S* component images of (a-b) of *HSI*.

In order to segment the boundary of wound bed, filtered *RGB* wound image was converted into *HSI* (*H*: hue, *S*: saturation, *I*: intensity) color space as it is more close to the way humans perceive the color. In fact, *H* describes pure color where *S* provides the degree to which a pure color is diluted by white light and *I* is subjective color [20]. In order to avoid any color conflict during segmentation of wound area from skin, only *S* component of *HSI* channels was selected here that showed the improved contrast at the wound boundary as shown in Figure 3.

### 2.3. Wound Area Segmentation Using Fuzzy Divergence Based Thresholding

Threshold selection is very crucial in wound area segmentation like any other images. The accuracy in segmentation is very often degraded due to overlapping intensities and pixel ambiguities especially at the junction between wound and nonwound (skin) regions. In order to reduce overlapping pixel intensity, a simple fuzzy divergence method was considered here to segment the wound area in *S* channel wound image [21, 22]. Ghosh et al. have proved fuzzy divergence based thresholding to be very much useful in medical image segmentation. For any grayscale image, pixel intensity ranges in [0, 255] where fuzzy image was defined by associating a membership value *μ*(*f*_*ij*_) to the intensity value *f*_*ij*_ at the (*i*, *j*)th pixel. And hence the fuzzy image was given as *A* = {*f*_*ij*_, *μ*(*f*_*ij*_)∣*μ* : *f*_*ij*_ → [0,1]}. Theoretically, for any two images *A* and *B* of same size *M* × *N* with distinct gray values, the information about the discrimination between the membership values at the (*i*, *j*)th pixel was given by Chaira and Ray as

(1) $$\begin{array}{c} \frac{e^{\mu_{A}(a_{ij})}}{e^{\mu_{B}(b_{ij})}}=e^{\mu_{A}(a_{ij})-\mu_{B}(b_{ij})}, \end{array}$$

where *i* = 0,1,…, *M* − 1 and *j* = 0,1,…, *N* − 1; *μ*_*A*_(*a*_*ij*_) and *μ*_*B*_(*b*_*ij*_) indicate the membership values of the (*i*, *j*)th pixel in images *A* and *B*, respectively [21]. Hence the discrimination of image *A* against *B* is

(2) IAB(μA,μB)=∑i=0M−1 ∑j=0 N−1[1−{1−μA(aij)}eμA(aij)−μB(bij)−μA(aij)eμB(bij)−μA(aij)].

Similarly, discrimination of image *B* against *A* is

(3) IBA(μA,μB)=∑i=0M−1 ∑j=0 N−1[1−{1−μB(bij)}eμB(bij)−μA(aij)−μB(bij)eμA(aij)−μB(bij)].

Hence, the total fuzzy divergence between *A* and *B* was computed by summing (1) and (2)

(4) D(μA,μB)=IAB(μA,μB)+IBA(μA,μB)=∑i=0M−1 ∑j=0 N−1[2−{1−μA(aij)+μB(bij)}eμA(aij)−μB(bij)−{1−μB(bij)+μA(aij)}eμB(bij)−μA(aij)].

Here membership values of pixel intensities of wound images in *S* channel were assessed using Gaussian membership function [22]. Thereafter, basic operations, namely, erosion and dilation in mathematical morphology, were used by using suitable structuring element for achieving boundary continuity preserving the wound tissue information. After getting the precise wound boundary, the only actual wound bed was segmented from the filtered (*RGB*) wound image for quantitative feature extraction.

### 2.4. Feature Extraction for Tissue Subclassification

In wound characterization, clinicians mainly target the distribution and density of the clinical features, namely, granulation, slough, and necrotic tissues, over wound bed. In order to provide the more accurate evaluation towards wound tissue classification, a set of quantitative color and textural features were computed here as these features provide useful information regarding color and microstructural descriptions of tissues.

#### 2.4.1. Color Features

Wound image analysis primarily deals with color information for clinical evaluation. Clinically, color bears significant information due to the properties of light passing/reflection through tissue. In view of this, we attempted here to extract color features quantitatively for understanding the wounds in various color spaces. In this work, fifteen color spaces, namely, *RGB*, *HSI*, *XYZ*, *Lab*, *Luv*, *LCH*, *HSV*, *HSL*, *YUV*, *YIQ*, CAT02 *LMS*, *YC*_*b*_ *C*_*r*_, JPEG-*YC*_*b*_ *C*_*r*_, *YD*_*b*_ *D*_*r*_, and *YP*_*b*_ *P*_*r*_ [19], having three color components in each color space were taken and hence total 45 color channels were considered for quantifying the color properties of individual tissues without considering spatial dependency between them. Original acquired wound image in *RGB* format was converted into other 14 color spaces by suitable conversion function [19]. Five color based features, namely, mean, standard deviation, skewness, kurtosis, and variance, were extracted from each of 45 color channels for every region of interest. Let *W*(*x*, *y*) be the segmented region of interest having total *N* number of pixels in the region then color features are computed as follows:

(5) Mean(μ)=1N∑i=1NWi(x,y),Standard  Deviation(σ)=1N∑i=1N(Wi(x,y)−μ)2,Variance(SN2)=1N∑i=1N(Wi(x,y)−μ)2,Skewness(S^)=  1N∑i=1N(Wi(x,y)−μσ)3,Kurtosis(K^)=1N∑i=1N(Wi(x,y)−μσ)4.

#### 2.4.2. Texture Features

**Figure 4:**
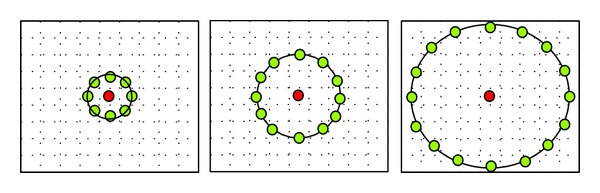
Neighborhood with different values of radius (*R*) for calculating LBP.

Texture represents microstructural information of self-similar pattern in a small region. Ten textural features, namely, Shannon's entropy [23], three local contrast features (based on mean, mode, and median), and six local binary pattern (LBP) features, were extracted from each of 45 color channels for every segmented wound region. Local contrast measures the variation in the pixel values of the given region with respect to a parameter chosen as a measure of the central tendency (mean, median, and mode) computed over the local region. Local contrast is computed as the difference between an average value of pixels above the central value and an average value of pixels below the central value. In view of this, three local contrast features were computed as follows:

(6) $$\begin{array}{c} \text{Local}\;\text{Contrast}\left(\text{LC}\right)=\frac{\left(T-A\right)}{\left(T+A\right)}, \end{array}$$

where *T* is the average value of all the pixels whose intensity values are equal to or greater than that of selected measure of the central tendency (mean, median, and mode) and *A* is a complementary average intensity value of remaining pixels which are less than the selected measure of the central tendency (mean, median, and mode). Local binary pattern (LBP) [24] is a simple and efficient method for textural analysis of gray scale images (see Figure 4). LBP labels the pixels of an image by thresholding the neighborhood of each pixel and considers the result as a binary number. LBP is a consolidating approach to the traditionally divergent structural and statistical models of textural analysis. Consider a sample neighborhood from the wound tissue region containing “*P*” pixels with *g*_*c*_ corresponding to the gray intensity value of the centre pixel. The texture *T* for this pixel is computed as

(7) $$\begin{array}{c} T=t\left(g_{c},g_{0},g_{1},\ldots ,g_{P-1}\right), \end{array}$$

where *g*_*p*_  (*p* = 0,…, *P* − 1) corresponds to the gray values of *P* equally spaced pixels on a circle of radius *R* as shown in Figure 3. The LBP for the centre pixel is computed by

(8) $$\begin{array}{c} {\text{LBP}}_{P,R}=\overset{P-1}{\underset{p=0}{\sum }}S\left(g_{p}-g_{c}\right)2^{p}, \end{array}$$

where

(9) $$\begin{array}{c} S\left(x\right)=\{\begin{array}{cc} 1, & x\geq 0,\\ 0, & x<0. \end{array} \end{array}$$

Rotation invariance is achieved by rotating the neighbor set clockwise such that maximal number of most significant bits is zero in the LBP code:

(10) $$\begin{array}{c} {\text{LBP}}_{P,R}^{ri}=\min\left\{\text{ROR}\left({\text{LBP}}_{P,R,i}\right)\;|\;i=0,1\ldots ,P-1\right\}. \end{array}$$

To include the local image texture contrast, a rotation invariant measure of local variance is given by

(11) $$\begin{array}{c} {\text{VAR}}_{P,R}=\frac{1}{P}\overset{P-1}{\underset{p=0}{\sum }}{\left(g_{p}-\mu \right)}^{2}, \end{array}$$

where

(12) $$\begin{array}{c} \mu =\frac{1}{P}\overset{P-1}{\underset{p=0}{\sum }}g_{p}. \end{array}$$

VAR_*P*,*R*_ was computed for three radius values as *R* = 1, 2 and 3 with the corresponding pixel count *P* being 8, 16, and 24, respectively. Mean of each of the LBP output image was calculated, which was combined with the mean local contrast of the image to obtain six features. Finally, a total of 675 features (5 color and 10 textural features on each of 45 color channels) were extracted. As the number of features was large, *F*-statistic [25] was used to find only the statistical significant features for classification.

### 2.5. Statistical Learning Schemes

Bayesian and support vector machine (SVM) being the most significant learning techniques were considered to learn the three types of wound tissues, namely, granulation, slough, and necrotic based on statistically significant color and texture features.

*(A) Bayesian Learning.* Bayesian classifier [26] was used for classifying three tissue types using significant color and texture features. Suppose there are *m* classes *C*_1_, *C*_2_, *C*_3_,…, *C*_*m*_, whereas *d* dimensional feature space *X* = (*x*_1_, *x*_2_,…, *x*_*d*_) is considered as wound tissue descriptors. For a particular feature set *X*, classifier predicts the tissue type in one of the three classes where it achieves higher posterior probability; that is, granulation pixel belongs to the class *C*_1_ if and only if *P*(*C*_*i*_∣*X*) > *P*(*C*_*j*_∣*X*) for 1 ≤ *j* ≤ *m*, *j* ≠ *i*. Posterior probability is obtained using Bayes' theorem as

(13) $$\begin{array}{c} P\left(C_{i}\;|\;X\right)=\frac{P\left(X\;|\;C_{i}\right)\cdot P\left(C_{i}\right)}{P\left(X\right)}, \end{array}$$

where *P*(*C*_*i*_) denotes the prior probability and total probability is defined as

(14) $$\begin{array}{c} P\left(X\right)=\overset{m}{\underset{i=1}{\sum }}P\left(X\;|\;C_{i}\right)P\left(C_{i}\right). \end{array}$$

*P*(*X*∣*C*_*i*_) indicates class-conditional probability. Under assumption of statistical independence, the joint likelihood function leads to product of marginal density functions, defined as

(15) P(X ∣ Ci)=∏k=1dP(xk ∣ Ci)=P(x1 ∣ Ci)×P(x2 ∣ Ci) ×P(x3 ∣ Ci)⋯×P(xd ∣ Ci).

The tissue pixel is classified into one class where it attains the maximum posterior probability. 

*(B) Support Vector Machine (SVM).* SVM is a well-known supervised learning technique which separates the classes more accurately especially for linearly nonseparable dataset [24]. In such situation, the input space of tissue pixels is transformed into a feature space using nonlinear function “Φ,” called kernel function. The feature space is a high-dimensional space in which the classes can be separated by a linear classifier. For instance, *d* dimensional feature vector *X* = (*x*_1_, *x*_2_,…, *x*_*d*_) describing pixel's characteristics is considered where tissue class level *y* was assigned to +1, −1. The discriminant function used in SVM with kernel function is found by *g*(*X*) = *W* ^*T*^Φ(*X*) + *b*. Here Φ(*X*) represents the mapping of input data *X* into the kernel space. Therefore, the optimization equation is found as

(16) $$\begin{array}{c} \text{Max.}\left[\overset{n}{\underset{i=1}{\sum }}\alpha_{i}-\overset{n}{\underset{i,j=1}{\sum }}\alpha_{i}\alpha_{j}y_{i}y_{j}\Phi {\left(x_{i}\right)}^{T}\Phi \left(x_{j}\right)\right]. \end{array}$$

**Figure 5:**
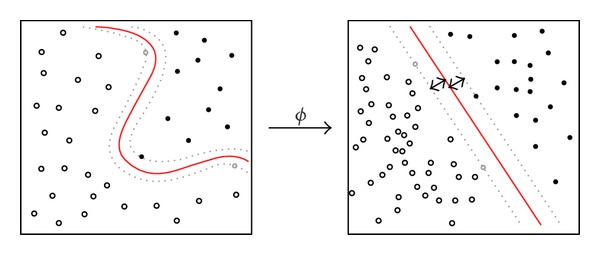
SVM based data classification.

Here, linear, 2nd and 3rd order polynomial and radial basis functions (RBF) were considered as kernel functions (see Figure 5) for classifying wound tissues into three classes [24].

*(C) Accuracy Computation and Statistical Validation Using Kappa Statistic.* Accuracy is computed for pixel prediction for three types of wound tissues using

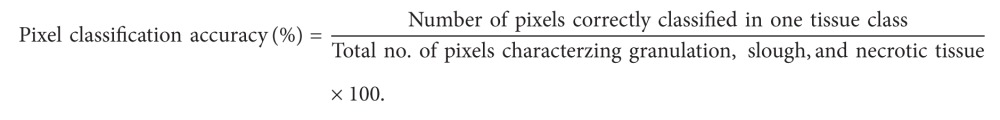
(17)
In addition, Cohen's Kappa statistics were used here to evaluate the agreement between expert and classifier based results towards tissue pixel classification [27].

## 3. Results

**Figure 6:**
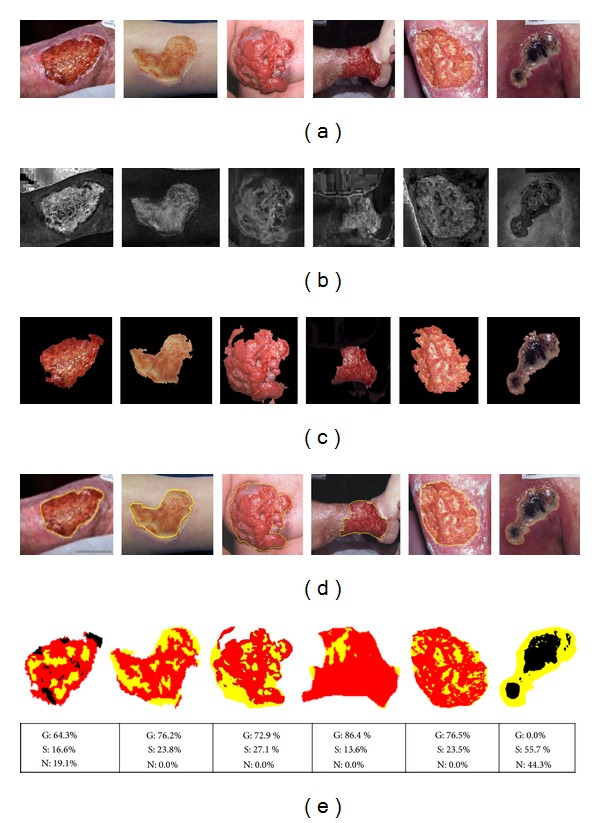
Segmented results of chronic wound areas using fuzzy divergence based thresholding: (a) original chronic wound images [burn, diabetic ulcer, malignant ulcer, pyoderma gangrenosum, venous ulcer, and pressure ulcer]; (b) saturation (*S*) component image under *HSI* color space transformation; (c) segmented wound areas; (d) ground truth marked by the clinician; (e) types of wound tissues (granulation, necrotic, and slough) characterized pseudocolored pixels; (e) representing % of granulation (*G*), slough (*S*), and necrotic (*N*) tissue pixels.

The median filtered wound images of *RGB* format (downloaded from Medetec Image Database) were transformed into *HSI* color space where “*S*” component images for different wounds were considered for segmentation. Thereafter, wound areas were segmented using fuzzy divergence based thresholding where Gaussian membership based divergence value for wound was 0.45 ± 0.02. The corresponding gray value for *S* component wound images was 207.4 ± 10.4. The machine generated segmented wound areas were also validated on ground truth images by clinical experts (Figure 5). From the wound database, medical experts identified total 767 tissue regions describing 222 regions as granulation tissue, 451 regions as slough tissue, and 94 regions as necrotic tissue based on 74 wound images. Five color and ten textural features were extracted for all the selected regions. Six local binary pattern (LBP) features, namely, LBP-1 for 8, LBP-2 for 16, and LBP-3 for 24 neighborhood points were computed. Out of the total 675 extracted features, 50 features were found to be statistically significant (*P* < 0.001) having *F*-value more than 21. Out of five color features only mean color value was significant. And mean values of LBP-1, LBP-2, and LBP-3 were the three features selected out of ten textural features form various color channels. Using selected features, wound tissues were classified into red granulation tissue, yellow slough tissue, and black necrotic tissue based on Bayesian and SVM classifiers. The proposed methodology was applied on various types of wound images and wound tissue pixels were recognized (Figure 6). For example, in case of the 1st image in Figure 6, red granulation, yellow slough, and black tissue were estimated as 64.3%, 16.6%, and 19.1%, respectively. From Table 1, it can be observed that Bayesian method provided 81.15% overall accuracy in predicting three types of tissue pixels.

SVM was used for the wound tissue pixel classification. Since the dataset was nonlinear in nature, different kernel functions have been used to obtain the suitable classifier. The confusion matrix shown in Table 2 denotes wound pixel classification matrix.

From Table 3, it can be observed that the overall accuracy of Bayesian classifier was 81.15% whereas tissue-wise accuracies were 86.84% for granulation tissue, 78.27% for slough, and 78.72% for necrotic tissue. By comparing various kernels for SVM, it may be suggested that SVM with 3rd polynomial kernel provided the highest overall accuracy, that is, 86.13% along with tissue-wise accuracies for all three wound tissues. The results were clinically validated with the ground truths where kappa statistic value was maximum (=0.793) for SVM with 3rd polynomial kernel in comparison with others including Bayesian approach. Wannous et al. also showed tissue classification scheme for reproducible wound assessment in telemedicine environments [28]. They have used one type of wound based on color and textures features. Here, six types of wound were considered to show the efficiency of our proposed method. Because of intrapixel ambiguities, the accuracy decreases while wound type increases. The proposed image processing methodology will assist a clinician to assess/monitor chronic wound's healing status through quantitative estimation of granulation, slough, and necrotic tissues in each type of wounds. In addition, it will also provide the wound area with augmented accuracy. Overall, the proposed method is able to generate efficient results in reproducible wound assessment. The paper is important in that it establishes novel technical guidelines for the evaluations of CW using relatively inexpensive technology. The proposed algorithms aid the identification of necrotic tissue within CWs in a very simple way especially in areas where there are few skilled wound care specialists. The proposed methodology requires relatively unsophisticated technology and is easily transmitted. It may assist in establishing further refinements to this technology.

## 4. Conclusion 

Summarizing, the present study attempted to develop a computer aided wound tissue classification scheme for chronic wound evaluation and management using wounds' images acquired through normal digital camera. The proposed methodology included computer vision and statistical pattern classification for automated characterization of three types of wound tissues, which are frequently required for diagnosis. The results showed SVM with 3rd degree polynomial provided highest classification accuracy for separating three types of wound tissue pixels based on color and texture features. Here we considered not only color features but also texture features. Moreover, it may be concluded that present findings could have important implications to the field of clinical evaluation and management of CW. Clinicians may now be provided with an objective, reliable, and efficient computational tool for segmentation and measurement of wound area facilitating an accurate assessment of wound healing, combining dimensional measurements with tissue characterization. Future work will include validation with larger data set of chronic wound images and the exploration of the performance of different segmentation and classification algorithms based on different color, textural, and statistical features. Moreover, this methodology may be directly extrapolated to other similar environments such as tissue segmentation on burn wound images or skin tumor pictures. Overall, our findings suggest that a computer aided wound assessment tool may assist health professionals to monitor the healing of CW during treatment. It may also provide clinical guidance through telewound care in remote areas where there is lack of clinical expert in wound management. This tool for wound assessment may also assist in computing real tissue areas by mapping tissue classification results on wound 3D surfaces.

## Figures and Tables

**Table 1:** Classification matrix of wound tissue pixels using Bayesian learning.

Original	Predicted pixels			Tissue-wise accuracy	Overall accuracy
	Granulation	Slough	Necrotic	(%)	
Granulation	192	21	9	86.48	81.15%
Slough	67	353	31	78.27	
Necrotic	5	15	74	78.72	

**Table 2:** Wound pixel classification matrix using SVM learning models.

SVM	Original	Predicted pixels		
		Granulation	Slough	Necrotic
Linear kernel	Granulation	184	35	3
	Slough	50	390	11
	Necrotic	14	34	46

2nd order polynomial	Granulation	182	33	7
	Slough	38	400	13
	Necrotic	5	22	69

3rd order polynomial	Granulation	195	23	4
	Slough	31	410	10
	Necrotic	3	16	75

RBF kernel	Granulation	184	32	6
	Slough	39	401	11
	Necrotic	4	20	70

**Table 3:** Performance evaluation of various classifiers for wound tissue classification.

Statistical learning schemes	Tissue-wise accuracy (%)			Overall accuracy (%)	Kappa statistic
	Granulation	Slough	Necrotic		
Bayesian classifier	86.48	78.27	78.72	81.15	0.704
SVM with linear kernel	82.88	86.47	48.93	72.76	0.653
SVM with 2nd polynomial kernel	81.98	88.69	73.40	81.35	0.718
SVM with 3rd polynomial kernel	**87.84**	**90.90**	**79.78**	**86.13**	**0.793**
SVM with RBF kernel	82.88	88.91	74.46	80.08	0.697
